# RNF12 X-Linked Intellectual Disability Mutations Disrupt E3 Ligase Activity and Neural Differentiation

**DOI:** 10.1016/j.celrep.2018.04.022

**Published:** 2018-05-08

**Authors:** Francisco Bustos, Anna Segarra-Fas, Viduth K. Chaugule, Lennart Brandenburg, Emma Branigan, Rachel Toth, Thomas Macartney, Axel Knebel, Ronald T. Hay, Helen Walden, Greg M. Findlay

**Affiliations:** 1 The MRC Protein Phosphorylation and Ubiquitylation Unit, School of Life Sciences, The University of Dundee, Dundee DD1 5EH, UK; 2 Institute of Molecular Cell and Systems Biology, The University of Glasgow, Glasgow G12 8QQ, UK; 3 Centre for Gene Regulation and Expression, School of Life Sciences, The University of Dundee, Dundee DD1 5EH, UK

**Keywords:** ubiquitin, protein ubiquitylation, E3 ubiquitin ligase, proteasomal degradation, RNF12/RLIM, intellectual disability, X-linked intellectual disability, embryonic stem cells, neural differentiation

## Abstract

X-linked intellectual disability (XLID) is a heterogeneous syndrome affecting mainly males. Human genetics has identified >100 XLID genes, although the molecular and developmental mechanisms underpinning this disorder remain unclear. Here, we employ an embryonic stem cell model to explore developmental functions of a recently identified XLID gene, the RNF12/*RLIM* E3 ubiquitin ligase. We show that RNF12 catalytic activity is required for proper stem cell maintenance and neural differentiation, and this is disrupted by patient-associated XLID mutation. We further demonstrate that RNF12 XLID mutations specifically impair ubiquitylation of developmentally relevant substrates. XLID mutants disrupt distinct RNF12 functional modules by either inactivating the catalytic RING domain or interfering with a distal regulatory region required for efficient ubiquitin transfer. Our data thereby uncover a key function for RNF12 E3 ubiquitin ligase activity in stem cell and neural development and identify mechanisms by which this is disrupted in intellectual disability.

## Highlights

• The RNF12 E3 ubiquitin ligase regulates stem cell differentiation to neurons
• RNF12/*RLIM* mutations in intellectual disability patients impair E3 ligase activity
• These mutations affect distinct RNF12 regions involved in catalysis
• RNF12/*RLIM* intellectual disability mutations drive abnormal neural differentiation

## Graphical Abstract

**Figure:**
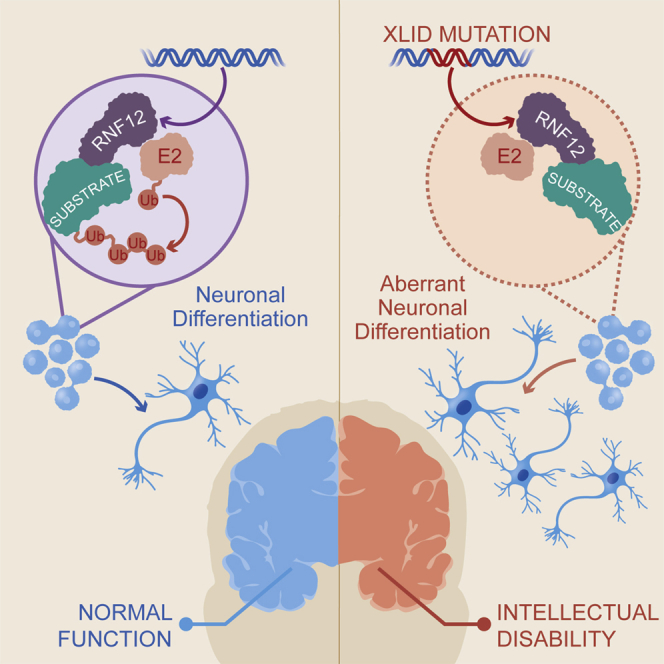

## Teaser

Bustos et al. show that the RNF12 E3 ubiquitin ligase regulates stem cell maintenance and neuronal differentiation. They demonstrate that RNF12/*RLIM* mutations identified in X-linked intellectual disability patients disrupt regions required for catalytic activity, which leads to compromised stem cell maintenance and abnormal neural differentiation.

## Introduction

Intellectual disability (ID) is a genetically heterogeneous and poorly understood developmental disorder, characterized by limited intellectual functioning and cognitive or adaptive behavior (van Bokhoven, 2011). It is estimated that ID affects 1%–2% of the world’s population. ID is characterized by abnormalities in various facets of neural development, including induction of neural differentiation genes, formation and establishment of neural projections, dendritic arborization, and dendritic spine morphology (Penzes et al., 2011, Telias and Ben-Yosef, 2014). Optimal kinetic and functional parameters of neural development are required for normal intellectual function, such that positive or negative deviation from these thresholds can lead to ID. For example, abnormal neural specialization (Telias and Ben-Yosef, 2014) and dendritic spine arborization (Iwase et al., 2016, Korb et al., 2017) are associated with ID syndromes. These and other neuropathological features of XLID have been modeled *in vitro* using pluripotent stem cells (Telias and Ben-Yosef, 2014).

At the molecular level, genome sequencing has identified many genes mutated in ID, including those with functions in transcription, chromatin, and cell signaling. Genes encoding enzymes involved in regulating post-translational modifications (PTMs) are frequently mutated in ID and related syndromes. These include the protein kinases DYRK1A (Luco et al., 2016), CDK16 (Deciphering Developmental Disorders Study, 2017), RSK2 (Delaunoy et al., 2001), and CASK (Hackett et al., 2010); the lysine demethylase KDM5C (Gonçalves et al., 2014); the E2 ubiquitin-conjugating enzyme UBE2A (Tsurusaki et al., 2017); the E3 ubiquitin ligases HUWE1 (Froyen et al., 2008), TRIP12 (Bramswig et al., 2017, Zhang et al., 2017), and MID2 (Geetha et al., 2014); and the deubiquitylases USP7 and OTUD6B (Santiago-Sim et al., 2017). Mutations in cell signaling regulators therefore represent an important class of ID, although the mechanisms by which disruptions in these components cause ID remain uncertain.

The X chromosome has been identified as an important genetic center for defining intellectual capacity. X-linked intellectual disability (XLID) represents a common subtype of ID, accounting for an estimated 16% of all male ID cases. Mutations in the X-linked E3 ubiquitin ligase RNF12/*RLIM* were reported in multiple XLID kindreds (Hu et al., 2016, Tønne et al., 2015), reinforcing the key role of protein ubiquitylation in intellectual development. RNF12 mutations are unique disease-linked X chromosome alterations that tightly segregate with affected males in XLID families (Hu et al., 2016, Tønne et al., 2015). RNF12 plays a critical role in processes with potential importance for intellectual development, including X chromosome inactivation (XCI) (Jonkers et al., 2009, Shin et al., 2010) and embryonic stem cell (ESC) differentiation (Zhang et al., 2012). However, the role of RNF12 in XLID and development of the nervous system has not been investigated.

Here, we show that RNF12/*Rlim* mutation in male ESCs accelerates induction of neural lineage markers and establishment of neurite outgrowths, a phenotype associated with ID. RNF12 E3 ubiquitin ligase activity supports the stem cell state to control ESC development, suggesting that RNF12 catalytic activity may be relevant for XLID pathogenesis. We show that RNF12/*RLIM* XLID mutants display impaired ubiquitylation of the key developmental RNF12 substrates REX1 and SMAD7. RNF12/*RLIM* XLID mutations disrupt E3 ligase activity via distinct mechanisms. RING domain mutants severely impair catalysis; mutations in a previously unappreciated basic regulatory region also interfere with ubiquitin transfer. Finally, we generate a knockin ESC model to confirm that RNF12 XLID mutation disrupts substrate degradation, stem cell maintenance, and neural differentiation. Altogether, these results map out a pathway whereby RNF12/*RLIM* mutations in XLID patients disrupt E3 ubiquitin ligase activity, leading to abnormal stem cell behavior and accelerated neural development characteristic of ID.

## Results

### RNF12/*Rlim* Gene Inactivation Promotes Abnormal Neural Development from Stem Cells

Enzymes in the ubiquitin system are frequently mutated in ID, suggesting a key role for protein ubiquitylation in neural development and pathogenesis of intellectual disorders. RNF12/*RLIM* was identified as an XLID gene, with point mutations closely segregating with the affected males from four XLID families (Hu et al., 2016, Tønne et al., 2015). RNF12 is implicated in aspects of early embryonic development, including XCI (Jonkers et al., 2009, Shin et al., 2010) and transforming growth factor β (TGF-β)/BMP (bone morphogenic protein) signaling (Zhang et al., 2012). We therefore hypothesized that RNF12 plays a key role in developmental processes that may be relevant for XLID.

Previous RNF12 studies were primarily conducted in female mice and *Rlim*−/− ESCs, which exhibit developmental alterations related to the critical role of RNF12 in XCI (Zhang et al., 2012). However, because XLID affects mainly males, we sought to explore how RNF12/*Rlim* gene inactivation in male ESCs affects developmental progression of stem cells toward lineages that make up the nervous system. Thus, we used CRISPR/Cas9 genome editing to generate RNF12/*Rlim* mutant male mouse ESCs (henceforth *Rlim* −/y ESCs). An introduced nonsense mutation is predicted to result in an RNF12 N-terminal truncation, although no protein is detected (Figure S1).

**Figure 1:**
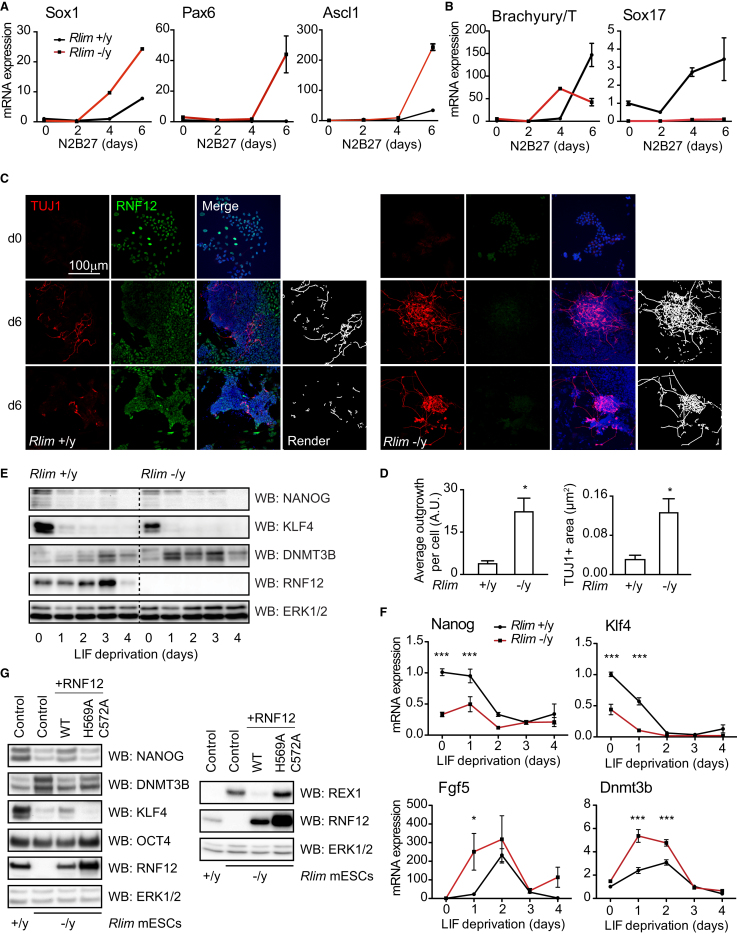
RNF12/*Rlim* Mutation Drives Accelerated Neural Differentiation and Aberrant ESC Pluripotency and Differentiation Signatures (A and B) *Rlim* +/y ESCs (black) and *Rlim* −/y ESCs (red) were cultured in N2B27 neural differentiation media for the indicated time and analyzed by qRT-PCR for relative mRNA expression of (A) Sox1, Pax6, and Ascl1 neural stem cell markers and (B) Brachyury/T and Sox17 mesodermal and endodermal markers, respectively. (C) *Rlim* +/y and −/y ESCs were cultured in N2B27 media for 0 or 6 days and stained with TUJ1 (red) and RNF12 (green) antibodies. Hoechst staining (blue) was used as a nuclear marker. Images were obtained as z stacks via confocal microscopy, and representative maximum-intensity z projections are shown. Renderings were generated using NeuriteTracer. (D) Quantification of average neurite outgrowth per cell (left panel) and TUJ1+ area normalized to nuclei area (right panel) from *Rlim* +/y and −/y ESCs (total cells counted: *Rlim* +/y = 12,273, *Rlim* −/y = 9,434). (E–G) *Rlim* +/y and *Rlim* −/y ESCs were subjected to LIF withdrawal for the indicated times. (E) NANOG, KLF4, DNMT3B, and RNF12 and ERK1/2 protein levels were determined by immunoblotting. (F) qRT-PCR analysis of *Nanog*, *Klf4*, *Fgf5*, and *Dnmt3b* mRNA levels in *Rlim* +/y ESCs (black) and *Rlim* −/y ESCs (red). (G) *Rlim* +/y or *Rlim* −/y ESCs were transfected with the indicated vectors, and NANOG, DNMT3B, KLF4, OCT4, RNF12, and ERK1/2 protein levels were determined by immunoblotting (left panel). *Rlim* +/y or *Rlim* −/y ESCs were transfected with the indicated vectors, and REX1, RNF12, and ERK1/2 protein levels were determined by immunoblotting (right panel). Data are represented as mean ± SD (n = 3). See also Figure S1.

Initially, we examined whether RNF12 is required for regulation of differentiation toward neural lineages. We employed the N2B27 differentiation system, which promotes efficient neural differentiation from ESCs (Ying et al., 2003). Following transfer into N2B27 media, *Rlim* +/y ESCs display a gradual increase in expression of the neural progenitor markers Sox1 and Pax6 and the key neural stem cell factor Ascl1 (Figure 1A). *Rlim* −/y ESCs display a rapid increase in Sox1, Pax6, and Ascl1 expression (Figure 1A), indicating that ESC differentiation to neural lineages is accelerated by RNF12/*Rlim* inactivation. Induction of the mesoderm marker Brachyury/T and endoderm marker Sox17 are diminished in *Rlim* −/y ESCs compared to wild-type controls (Figure 1B). Thus, RNF12 modulates ESC development toward mesendodermal and neuroectodermal fates.

We next investigated whether the accelerated induction of neural progenitor markers observed in differentiating *Rlim* −/y ESCs is accompanied by morphological hallmarks of neuronal development. By day 6 of N2B27 differentiation, few TUJ1+ neurite outgrowths are observed in *Rlim* +/y ESCs (Figure 1C). In contrast, *Rlim* −/y ESCs at day 6 frequently display an elaborate network of TUJ1+ neurite outgrowths, in addition to TUJ1 staining in the cell body (Figure 1C). This observation is confirmed by computational rendering to quantitatively determine the length and area of TUJ1+ neurite outgrowths (Figure 1D). Differentiating *Rlim* −/y ESCs thus acquire transcriptional and morphological characteristics of neuronal cells in advance of control *Rlim* +/y ESCs. These data indicate that RNF12 controls developmental timing during neuronal differentiation, suggesting a potential function of RNF12 that may be disrupted in XLID.

### RNF12/*Rlim* E3 Ligase Inactivation Leads to Loss of Stem Cell Identity

Because RNF12 is implicated in stem cell regulation (Jonkers et al., 2009, Shin et al., 2010, Zhang et al., 2012), we hypothesized that RNF12 controls neural differentiation by regulating expression of ESC-specific genes. We therefore investigated the effect on RNF12/*Rlim* gene inactivation on expression of the pluripotency gene regulatory network. When cultured in leukemia inhibitory factor (LIF) and fetal calf serum (FCS), *Rlim* +/y ESCs express high levels of key naive pluripotency factors NANOG and KLF4, in contrast to *Rlim* −/y ESCs (Figures 1E and 1F). *Rlim* −/y ESCs are more responsive to differentiation induced by LIF withdrawal, as evidenced by rapidly increased expression of DNMT3B/*Dnmt3b* (Figures 1E and 1F), a factor associated with lineage priming and differentiation, and *Fgf5* (Figure 1F), an early marker of ESC priming toward the epiblast lineage. These data indicate that RNF12/*Rlim* gene inactivation promotes exit from the stem cell state toward a lineage-primed state.

We then determined whether RNF12 E3 ligase activity is required to maintain expression of stem cell-specific genes. To this end, we transfected *Rlim* −/y ESCs with either the wild-type mouse RNF12 or an E3 ubiquitin ligase-defective mutant of RNF12 (H569A/C572A). The NANOG/KLF4/DNMT3B gene signature observed in *Rlim* −/y ESCs (Figures 1E and 1F) is rescued by expression of wild-type RNF12, but not RNF12 H569A/C572A (Figure 1G, left panel). In contrast, OCT4 levels are unaffected by RNF12 E3 ligase activity (Figure 1G, left panel). As expected, degradation of REX1, which is ubiquitylated by RNF12 to initiate imprinted XCI (Gontan et al., 2012), requires RNF12 catalytic activity (Figure 1G, right panel). RNF12 E3 ligase activity is therefore required for maintenance of stem cell-specific genes to suppress differentiation. Mutational inactivation of RNF12 thus alters the developmental potential of stem cells, leading to abnormal neural marker induction and acquisition of neuronal cell morphology.

### Characterization of RNF12 Mutations Identified in XLID Patients

**Figure 2:**
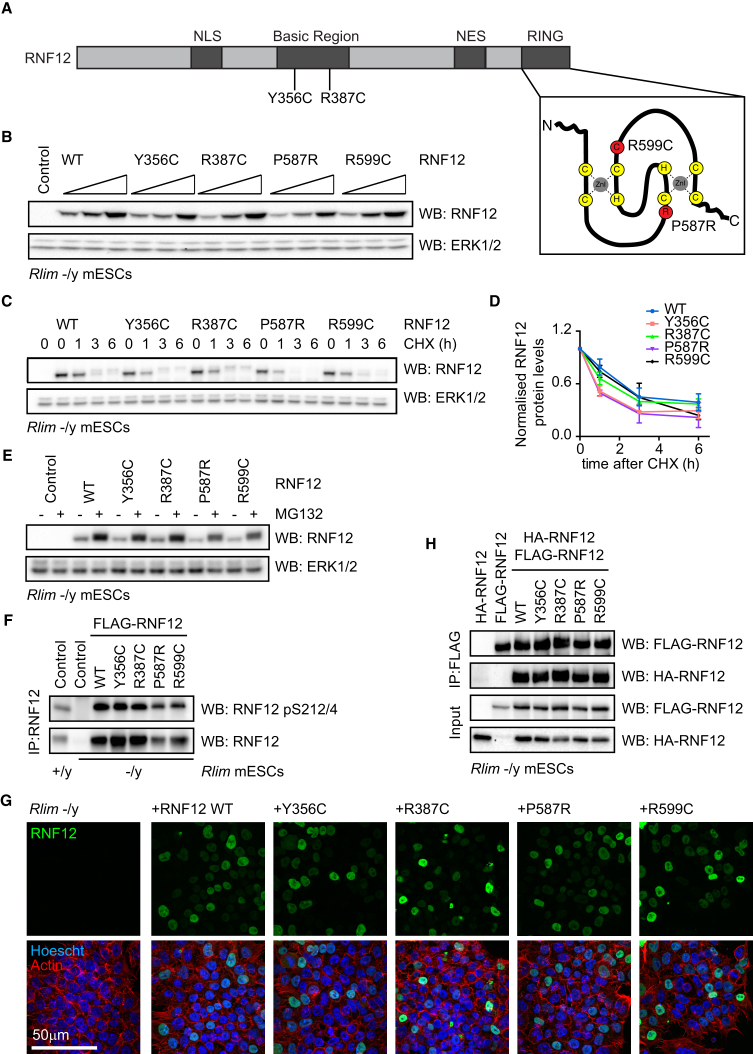
RNF12 Protein Stability, Dimerization, and Nuclear Localization Are Unaltered by XLID-Associated Mutations (A) Schematic of RNF12 indicating location of XLID mutations. NLS, nuclear localization signal; NES, nuclear export signal. Inset: positions of XLID mutations (red circles) within the RNF12 RING domain. Adapted from Metzger et al. (2014). (B) *Rlim* −/y ESCs were transfected with WT human RNF12 or XLID mutants, and RNF12 and ERK1/2 levels were determined by immunoblotting. (C) *Rlim* −/y ESCs were transfected with WT RNF12 or XLID mutants and treated with 350 μM cycloheximide (CHX) for the indicated times. RNF12 and ERK1/2 levels were determined by immunoblotting. (D) Quantification of western blot signals shown in (C). Data are represented as mean ± SEM (n = 3). (E) *Rlim* −/y ESCs were transfected with indicated vectors and treated with 10 μM MG132 for 6 hr. RNF12 and ERK1/2 levels were determined by immunoblotting. (F) *Rlim* +/y or *Rlim* −/y ESCs were transfected with the indicated constructs, and RNF12 NLS phosphorylation at Ser212/Ser214 and total RNF12 were analyzed by immunoblotting. (G) ESCs treated as in (F) were fixed, and subcellular localization of WT RNF12 or XLID mutants was analyzed via immunofluorescence and confocal microscopy. (H) *Rlim* −/y ESCs were transfected with N-terminal FLAG- or HA-tagged WT RNF12 or XLID mutants and lysates subjected to FLAG co-immunoprecipitation and immunoblot analysis with FLAG or HA antibodies. See also Figure S2.

Our data implicating RNF12 E3 ubiquitin ligase activity in stem cell maintenance and neuronal differentiation suggest that patient-derived XLID mutations may disrupt RNF12 catalytic activity. P587R and R599C XLID mutants reside within the RING domain of RNF12 that is responsible for E3 ubiquitin ligase catalytic activity (Figure 2A) (Das et al., 2013, Hodson et al., 2014, Joazeiro et al., 1999). Y356C and R387C lie in a basic stretch reportedly involved in RNF12 substrate binding (Figure 2A) (Gontan et al., 2012, Ostendorff et al., 2002). We therefore sought to elucidate the effect of XLID-associated mutations on RNF12.

To functionally characterize RNF12/*RLIM* XLID mutants, we developed a strategy to replace endogenous RNF12 in ESCs. When expressed in *Rlim* −/y ESCs, human RNF12 and XLID mutants are expressed at comparable levels (Figure 2B). XLID mutations in other genes have been found to disrupt protein stability, exemplified by *KDM5C* (Brookes et al., 2015). However, when expressed in *Rlim* −/y ESCs treated with cycloheximide, wild-type human RNF12 and RNF12 XLID mutant proteins display a similar rate of degradation (Figures 2C and 2D), suggesting that these mutations do not have a major impact on RNF12 protein stability. Proteasomal inhibition stabilizes wild-type RNF12 and XLID mutants to a similar extent (Figure 2E), indicating that these proteins are turned over at a similar rate. Our data therefore argue that RNF12 stability is unlikely to underpin pathogenesis of patient-derived RNF12 XLID mutants.

### RNF12 XLID Mutants Localize to the Nucleus and Dimerize

We next determined the effect of XLID point mutants on RNF12 regulation. RNF12 nuclear translocation is required for function and is driven by phosphorylation of a nuclear localization signal (NLS) (Figure 2A) motif by an unidentified kinase (Jiao et al., 2013). RNF12 XLID mutants are phosphorylated at Ser212/214 within this motif to a similar level as wild-type human RNF12 (Figure 2F). We also examined sub-cellular localization of RNF12 XLID mutants. Wild-type human RNF12 expressed in *Rlim* −/y ESCs localizes to the nucleus as expected (Figure 2G), similar to endogenous mouse RNF12 (Figure S2). RNF12 XLID mutants also localize to the nucleus in a manner similar to wild-type RNF12 (Figure 2G). Finally, because RING E3 ligases frequently function as dimers (Metzger et al., 2014), we addressed whether RNF12 XLID mutations disrupt RNF12 dimerization. When expressed in *Rlim* −/y ESCs, FLAG-tagged wild-type RNF12 co-immunoprecipitates with hemagglutinin (HA)-tagged wild-type RNF12 (Figure 2H), confirming that RNF12 dimerizes or self-associates *in vivo*. RNF12 XLID mutants self-associate to a similar level as wild-type RNF12 (Figure 2H), indicating that dimerization is not disrupted by RNF12 XLID mutations. Altogether, our data show that RNF12 XLID mutants are essentially indistinguishable from wild-type RNF12 in terms of stability, regulation of subcellular localization, and dimerization.

### XLID Mutations Impair RNF12 Substrate Ubiquitylation and Degradation *In Vivo*

**Figure 3:**
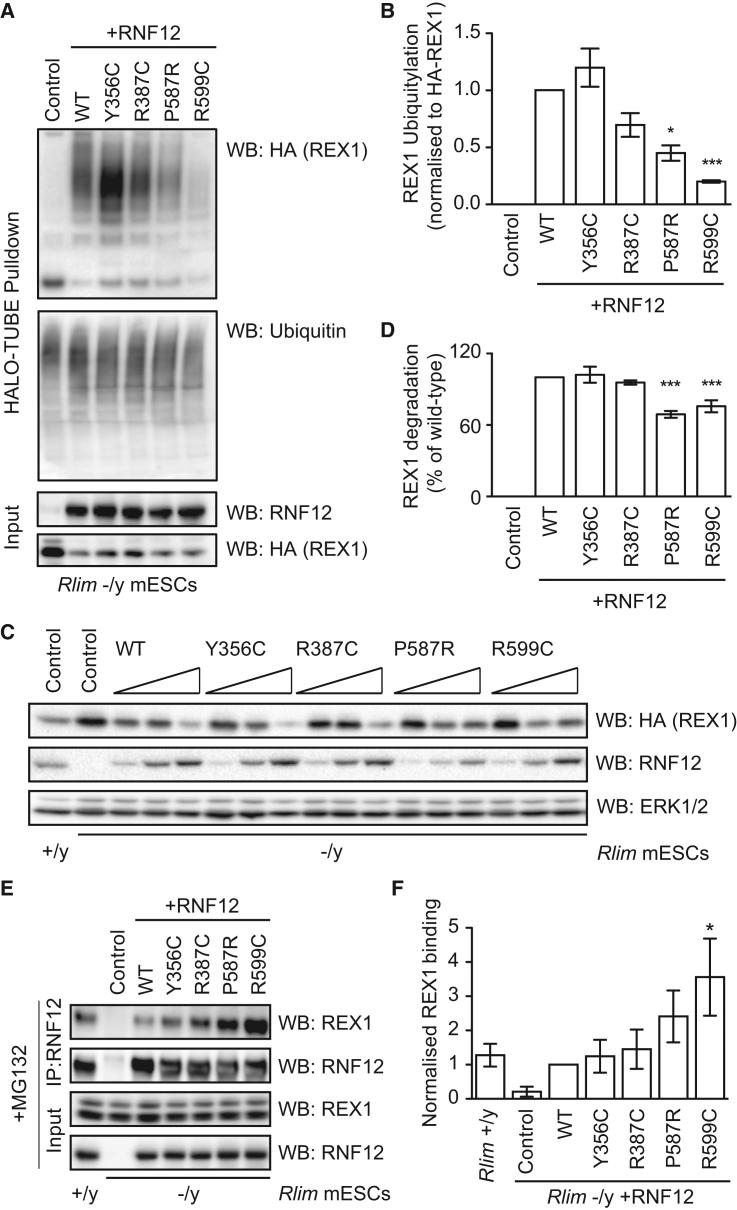
RNF12 XLID Mutations Disrupt Substrate Processing *In Vivo* (A) *Rlim* −/y ESCs were transfected with HA-REX1 and WT RNF12 or XLID mutants. Ubiquitylated proteins were captured using HALO-TUBE resin, and HA-REX1 ubiquitylation was determined by immunoblotting. Total ubiquitin levels are shown as a control. (B) Quantification of HA-REX1 ubiquitylation shown in (A) was normalized to the HA-REX1 input signal. (C) *Rlim* +/y or −/y ESCs were transfected with HA-REX1 and WT RNF12 or XLID mutants, and HA-REX1 and ERK1/2 levels were determined by immunoblotting. (D) Quantification of HA-REX1 signal intensities for RNF12 XLID mutants relative to WT RNF12 shown in (C). (E) *Rlim* +/y or −/y ESCs were transfected with the indicated vectors, treated with 10 μM MG132 for 6 hr before RNF12 immunoprecipitation. REX1 and RNF12 levels were determined by immunoblotting. (F) Quantification of REX1 interaction with RNF12 XLID mutants relative to WT RNF12. Data are represented as mean ± SEM (n = 3). HA-REX1 ubiquitylation in (A) and degradation in (C) were determined at 48 and 24 hr post-transfection, respectively. See also Figure S3.

These findings prompted us to investigate the impact of RNF12 XLID mutations on substrate processing. RNF12 controls X-linked gene expression by ubiquitylating the REX1 transcription factor to initiate imprinted XCI (Gontan et al., 2012), suggesting that REX1 may be a relevant RNF12 target in ID. REX1 ubiquitylation is essentially undetectable in *Rlim* −/y ESCs (Figure 3A) but is induced by 48 hr expression of human RNF12, as measured by specific capture of ubiquitylated HA-REX1 on a tandem ubiquitin binding element (TUBE) (Figure 3A). We show that HA-REX1 ubiquitylation and degradation depend on RNF12 E3 ubiquitin ligase activity in these assays (Figure S3). RNF12 XLID RING domain mutants also display significantly impaired REX1 ubiquitylation compared to wild-type human RNF12 (Figures 3A and 3B). However, XLID mutants in the basic region retain the ability to promote REX1 ubiquitylation (Figures 3A and 3B), suggesting that a subset of RNF12 XLID mutants disrupts substrate ubiquitylation *in vivo*.

We then addressed whether RNF12 XLID mutants alter REX1 proteasomal degradation. In *Rlim* −/y ESCs, HA-REX1 protein is stabilized and highly expressed when compared to *Rlim* +/y ESCs (Figure 3C). Expressing human RNF12 for 24 hr in *Rlim* −/y ESCs restores REX1 degradation (Figure 3C). In contrast, RNF12 P587R and R599C mutants in the RING domain are significantly impaired for REX1 degradation (Figures 3C and 3D), consistent with reduced REX1 ubiquitylation (Figures 3A and 3B). RNF12 XLID mutants Y356C and R387C in the basic region retain the ability to promote REX1 ubiquitylation (Figures 3A and 3B) and degradation (Figures 3C and 3D). Altogether, our results indicate that although all RNF12 XLID mutants retain some E3 ubiquitin ligase activity, a subset of mutations within the RING domain significantly impair REX1 substrate ubiquitylation and degradation. These data suggest that defective RNF12 substrate processing is a potential mechanism underpinning XLID.

### RNF12 XLID Mutants Display Impaired E3 Ubiquitin Ligase Catalytic Activity

Our findings prompted us to investigate the mechanisms by which XLID mutations affect RNF12-dependent ubiquitin transfer. First, we examined whether RNF12 XLID mutants alter substrate engagement, because both the RING domain and the basic region mutated in XLID are reportedly involved in RNF12 substrate recruitment (Gontan et al., 2012, Ostendorff et al., 2002). Following treatment with the proteasomal inhibitor MG132, REX1 efficiently co-immunoprecipitates with wild-type RNF12 (Figure 3E). RNF12 Y356C and R387C interact with REX1 to a similar level as wild-type RNF12 (Figures 3E and 3F). RNF12 RING domain mutants P587R and R599C show enhanced REX1 substrate binding (Figures 3E and 3F), suggesting that RNF12 RING domain mutants trap REX1 substrate in a nonproductive complex. Substrate trapping by an RNF12 RING mutant has previously been described and exploited to identify SMAD7 as an RNF12 substrate (Zhang et al., 2012). These results indicate that although RNF12 XLID mutants display impaired REX1 substrate ubiquitylation and degradation, this is not explained by defective substrate engagement.

A subset of RNF12 XLID mutations that alter substrate modification *in vivo* is found in the catalytic RING domain and therefore may interfere with catalytic E3 ubiquitin ligase activity. We sought to directly test this possibility *in vitro*. Because E3 ubiquitin ligase activity can vary depending on the E2-conjugating enzyme used (Christensen et al., 2007), we systematically determined the E2 or E2s that function with RNF12. A ubiquitin chain elongation assay of recombinant RNF12, in combination with fluorescently labeled ubiquitin (Ub-IR^800^), UBE1 E1, and a panel of 31 E2 enzymes, identified UBE2D and UBE2E families and UBE2W as major RNF12 partner E2-conjugating enzymes (Figure S4A). Of those, RNF12-UBE2D shows the highest chain-elongating activity (Figure S4A).

**Figure 4:**
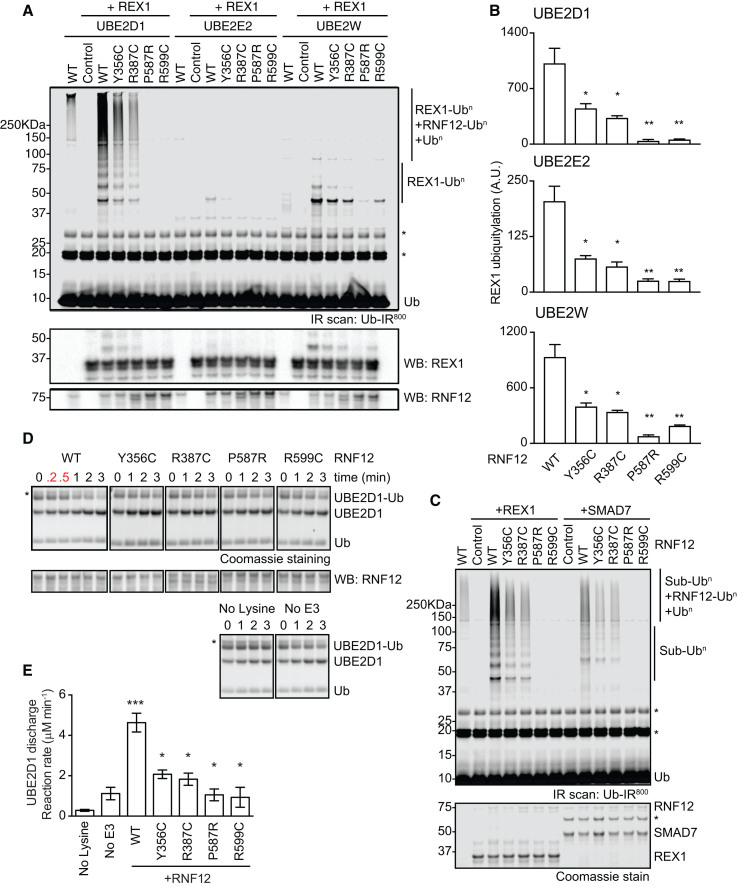
RNF12 XLID Mutants Display Impaired E3 Ubiquitin Ligase Catalytic Activity (A) *In vitro* REX1 substrate ubiquitylation assay of WT RNF12 and XLID mutant proteins using UBE2D1, UBE2E2, and UBE2W E2-conjugating enzymes. Fluorescently labeled ubiquitylated proteins were detected by infrared scan (Ub-IR^800^), and REX1 and RNF12 levels were determined by immunoblotting. Specific ubiquitylated REX1 (REX1-Ub^n^) and RNF12 (RNF12-Ub^n^) signals are indicated. ^∗^Nonspecific fluorescent band. (B) Quantification of REX1 ubiquitylation shown in (A). (C) *In vitro* REX1 and SMAD7 ubiquitylation assays performed as in (A) (Ub-IR^800^, top panel). REX1 and SMAD7 levels were determined by Coomassie staining (bottom panel). ^∗^Nonspecific band. (D) RNF12 XLID mutant proteins are impaired for UBE2D1 E2 ubiquitin discharge. Coomassie-stained gels showing RNF12-mediated ubiquitin (Ub) discharge from pre-formed UBE2D1 conjugates (UBE2D1-Ub). ^∗^Nonspecific band. RNF12 levels were determined by immunoblotting. (E) Determination of E2 discharge reaction rate from signal intensities obtained in (D). Data are represented as mean ± SEM (n = 3). See also Figure S4.

We then sought to investigate whether RNF12 XLID mutants alter substrate ubiquitylation by RNF12-E2 pairs *in vitro*. In the first instance, we used REX1 as a model RNF12 substrate. RNF12-UBE2D1 catalyzes efficient REX1 substrate ubiquitylation, in addition to background ubiquitin chain elongation (Figures 4A and S4B). XLID mutants P587R and R599C in the RING domain of RNF12 display severely compromised REX1 ubiquitylation (Figures 4A, 4B, and S4B), whereas RNF12 XLID mutations Y356C and R387C in the basic region display reduced but less severe defects in REX1 substrate ubiquitylation (Figures 4A, 4B, and S4B). Similar results are obtained with RNF12-UBE2E2 or RNF12-UBE2W pairs (Figures 4A and 4B), although RNF12-UBE2W primarily mono-ubiquitylates REX1 (Figure 4A). Our data therefore demonstrate that XLID mutations interfere with RNF12 E3 ubiquitin ligase activity, independent of the partner E2.

An important question is whether RNF12 XLID mutants disrupt ubiquitylation of other RNF12 substrates. In addition to REX1, RNF12 ubiquitylates the TGF-β/BMP pathway regulator SMAD7 in ESCs (Zhang et al., 2012). We confirm that RNF12 directly ubiquitylates SMAD7 *in vitro*, and this activity is disrupted by XLID mutations to a similar extent to that previously observed for REX1 ubiquitylation (Figure 4C). These data indicate that RNF12 XLID mutations disrupt ubiquitylation of multiple substrates, strongly suggesting that effects of RNF12 XLID mutations are unlikely to be substrate specific. Altogether, our results demonstrate that XLID mutations in the catalytic RING domain and the basic region disrupt RNF12 substrate ubiquitylation, albeit with different potency.

### RNF12 XLID Mutants Disrupt Ubiquitin Discharge from a Partner E2-Conjugating Enzyme

A key facet of RING E3 ligase activity is the ability to catalyze ubiquitin transfer from a ubiquitin-loaded E2-conjugating enzyme to free lysine. Because these assays are substrate independent, we investigated whether RNF12 XLID mutants interfere with E2 ubiquitin discharge. As expected, wild-type RNF12 drives ubiquitin discharge from ubiquitin-loaded UBE2D1 (Figures 4D and 4E). RNF12 XLID mutants in both the catalytic RING domain and the basic region are impaired in their ability to promote E2 ubiquitin discharge (Figures 4D and 4E). This is consistent with the key role of the RING domain in ubiquitin transfer by an RNF12-E2 complex. However, these results also uncover a previously unappreciated role for the RNF12 basic region in promoting efficient ubiquitin transfer from an E2-conjugating enzyme. Therefore, XLID patient-derived RNF12 mutations disrupt E3 ubiquitin ligase catalytic activity by interfering with distinct functional regions.

### RNF12 XLID Knockin ESCs Confirm Defects in RNF12 Substrate Degradation, Stem Cell Maintenance, and Neural Differentiation

**Figure 5:**
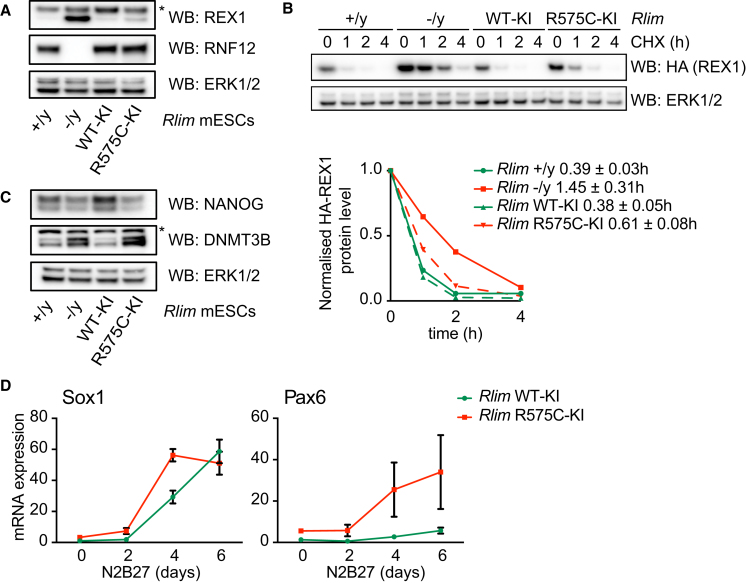
CRISPR/Cas9 Knockin RNF12 XLID Mutant ESCs Display Defects in Substrate Degradation and Abnormal Differentiation (A) *Rlim* WT and XLID knockin ESCs (WT-KI and R575C-KI) (see Figure S5 for details) were analyzed for REX1, RNF12, and ERK1/2 levels by immunoblotting. (B) *Rlim* +/y (green line), *Rlim* −/y, *Rlim* WT-KI, and *Rlim* R575C-KI ESCs were transfected with HA-REX1 and treated with 350 μM cycloheximide (CHX) for the indicated times. HA-REX1 and ERK1/2 levels were determined by immunoblotting (top panel). Quantification of HA-REX1 signal intensities determined in (B) was used to calculate HA-REX1 protein half-life (h) in the indicated ESC lines. (C) *Rlim* WT-KI and *Rlim* R575C-KI ESCs were cultured in absence of LIF, and NANOG, DNMT3B, and ERK1/2 levels were determined by immunoblotting. (D) *Rlim* WT-KI and *Rlim* R575C-KI ESCs were cultured in N2B27 media for the indicated times, and Sox1 and Pax6 mRNA expression was determined by qRT-PCR. Data are represented as mean ± SD (n = 3). ^∗^Nonspecific band. See also Figure S5.

We next sought to directly address the impact of endogenous RNF12/*Rlim* XLID mutation on RNF12 substrate processing and stem cell differentiation using a CRISPR/Cas9 knockin strategy. We generated paired control *Rlim* wild-type knockin (WT-KI) and *Rlim* R575C (the mouse equivalent of the human RNF12 R599C XLID mutation) knockin (R575C-KI) ESC lines (Figure S5). *Rlim* R575C/y ESCs display increased REX1 substrate expression when compared to *Rlim* WT-KI ESCs (Figure 5A). This is caused by reduced REX1 ubiquitylation and degradation, because REX1 displays increased half-life in *Rlim* R575C/y ESCs (0.61 ± 0.08 hr) when compared to *Rlim* WT-KI ESCs (0.38 ± 0.05 hr) (Figure 5B). The effect of RNF12 XLID knockin (*Rlim* R575C/y) is less dramatic than that of gene knockout (*Rlim* −/y) (Figures 5A and 5B), likely as a result of noncatalytic functions of RNF12 in REX1 processing (Wang et al., 2017). However, these data demonstrate that XLID mutation of the endogenous RNF12/*Rlim* gene locus disrupts substrate degradation.

Finally, we explored functional effects of RNF12 XLID mutation on ESC maintenance and neural differentiation. When cultured in the absence of LIF, *Rlim* R575C/y ESCs display reduced NANOG and increased DNMT3B expression compared to *Rlim* WT-KI ESCs (Figure 5C). We thus tested induction of neural lineage markers following ESC differentiation in N2B27 media. When compared to *Rlim* WT-KI ESCs, *Rlim* R575C/y ESCs display increased expression of neural markers Sox1 and Pax6 at early time points (Figure 5D). These data indicate that RNF12 XLID mutation accelerates ESC neural differentiation, echoing our previous observations (Figure 1). As observed previously, the RNF12 XLID knockin (*Rlim* R575C/y) mutation phenotype is less dramatic than RNF12/*Rlim* gene knockout. Nevertheless, our results confirm that RNF12 mutations found in XLID patients disrupt substrate ubiquitylation, driving aberrant stem cell differentiation and neuronal specification.

## Discussion

In this paper, we show that RNF12/*RLIM* XLID mutations disrupt RNF12 E3 ligase activity and that RNF12 catalytic function regulates differentiation of stem cells to neural cells. Our data provide a framework to understand how RNF12/RLIM mutations cause XLID and shed light on potential mechanisms that regulate development of the nervous system. Our findings also provide further evidence of the importance of PTMs such as the protein ubiquitylation system in ID.

In this regard, we elucidate biochemical mechanisms by which XLID mutations disrupt protein ubiquitylation by RNF12. We show that XLID mutations impair RNF12 ubiquitin transfer by interfering with distinct functional regions. RING domain mutants markedly diminish RNF12 E3 ligase activity both *in vitro* and in cells, while mutation of a previously unappreciated accessory basic region of RNF12 also disrupts catalysis. However, RNF12 basic region mutations are less potent and were uncovered only by highly sensitive initial rate measurement of RNF12 substrate ubiquitylation and E2 ubiquitin discharge *in vitro*. This facet of the RNF12 catalytic mechanism is reminiscent of the CBL-B RING E3 ubiquitin ligase, which uses a non-RING priming element to ensure optimized ubiquitin transfer (Dou et al., 2013). However, detailed structural studies are required to fully characterize the role of the RNF12 basic region in catalysis.

A key question concerns the mechanism by which RNF12 basic region mutations cause XLID. Although our biochemical data demonstrate that RNF12 XLID mutants are significantly impaired for E3 ubiquitin ligase activity independent of E2 or substrate, we observe significant REX1 substrate processing by RNF12 XLID mutants *in vivo*. This discrepancy could be explained by observations showing RNF12 also promotes REX1 degradation via alternative mechanisms, e.g., recruiting other E3 ligases (Wang et al., 2017). RNF12 XLID mutants may therefore lack E3 ligase activity while retaining other properties required for REX1 regulation. Whether RNF12 XLID mutants affect processing of substrates that function in neural development is not known. However, we observe impaired RNF12 E3 ligase activity toward at least two RNF12 substrates, which indicates that RNF12 XLID mutations are likely to affect RNF12 substrate processing in a variety of developmental contexts. Furthermore, mutations that subtly alter biochemical function, such as RNF12 XLID mutations in the basic region, may, over the timescale of nervous system development, have a cumulative impact leading to XLID. In support of this notion, very weak activating mutations in genes encoding components of the RAS-ERK1/2 mitogen-activated protein kinase (MAPK) signaling pathway can cause the developmental disorder known as Noonan syndrome (Roberts et al., 2007, Tartaglia et al., 2007).

Finally, we employ a stem cell model system to show that RNF12/*Rlim* gene disruption and introduction of an XLID mutation in male ESCs affect stem cell maintenance and kinetics of neural development. This phenotype has been described in other models of ID (Telias and Ben-Yosef, 2014, Telias et al., 2013), thereby implicating deregulated neural development, differentiation, and function as potentially important pathogenic mechanisms in XLID. We provide evidence that deregulated stem cell maintenance and specification of neural progenitors are observed by RNF12/*Rlim* deletion or XLID mutation. Further investigation into the function of RNF12 in regulating the stem cell state is required to fully understand its role in XLID. In this regard, the stem cell-specific factor NANOG controls RNF12 mRNA expression (Navarro et al., 2011), while paradoxically, our data show that RNF12 promotes NANOG expression. This tight cross-regulation may be important to prevent XCI in pluripotent cells while promoting ESC differentiation once RNF12 expression is extinguished following XCI. This system would thereby ensure that XCI is closely coupled to the onset of differentiation. Future research will also seek to address how RNF12/*Rlim* mutations affect mammalian neural development and explore the molecular functions of RNF12 in development of the nervous system by identifying RNF12 substrates whose ubiquitylation is required for correct neural specification and neuronal function.

## Experimental Procedures

### Cell Culture

WT male mouse ESCs (CGR8 line) were cultured in 0.1% gelatin (w/v)-coated plates in DMEM containing 10% FCS (v/v), 5% knockout serum replacement (v/v), 2 mM glutamine, 0.1 mM minimum essential media (MEM) nonessential amino acids, 1 mM sodium pyruvate, and penicillin and streptomycin (all from Thermo Fisher Scientific); 0.1 mM β-mercaptoethanol (Sigma-Aldrich); and 100 ng/mL LIF in a controlled atmosphere at 5% CO_2_ and 37°C. After 24 hr, transfected cells were selected with 2 μg/mL puromycin and grown for further 24 hr. For differentiation assays, cells were cultured in absence of LIF for up to 4 days. For neural differentiation assays, cells were cultured in N2B27 media composed of 1% B27 supplement (v/v), 0.5% N2 supplement (v/v), and 2 mM glutamine (all from Thermo Fisher Scientific); 0.1 mM β-mercaptoethanol (Sigma-Aldrich); and penicillin and streptomycin in 1:1 DMEM/F12:Neurobasal medium (both from Thermo Fisher Scientific). The media was changed every 48 hr. For protein synthesis or proteasome inhibition, cells were treated with 350 μM cycloheximide or 10 μM MG132 (both from Sigma-Aldrich), respectively.

### Transfection and Plasmids

ESCs were transfected with Lipofectamine LTX (Thermo Fisher Scientific) according to the manufacturer’s instructions. Plasmids used in this study generated by the Division of Signal Transduction and Therapy, University of Dundee, in pCAGGS puro backbone were mouse RNF12 (DU50610), RNF12 H569A C572A (DU50631), and HA-REX1 (DU58024); human RNF12 (DU53765), RNF12 Y356C (DU53771), RNF12 R387C (DU53772), RNF12 P587R (DU53776), and RNF12 R599C (DU53773); human 3FLAG RNF12 (DU53879), RNF12 Y356C (DU53880), RNF12 R387C (DU53881), RNF12 P587R (DU53882), and RNF12 R599C (DU53883); and human HA RNF12 (DU53884), RNF12 Y356C (DU53885), RNF12 R387C (DU53886), RNF12 P587R (DU53887), and RNF12 R599C (DU53888). Plasmids generated in pGEX6P1 backbone for expression in BL21 *E. coli* were human RNF12 (DU53876), RNF12 R387C (DU53877), RNF12 P587R (DU53878), RNF12 Y356C (DU53897), and RNF12 R599C (DU53898) and mouse REX1 (DU53244). All cDNA clones can be found at the Medical Research Council Protein Phosphorylation and Ubiquitin Unit (MRC-PPU) Reagents and Services website http://mrcppureagents.dundee.ac.uk/.

### CRISPR/Cas9 D10A Genome Editing

For generation of *Rlim* −/y ESCs, we transfected WT ESCs with pX335 and pKN7 vectors (Addgene) containing the guide RNA (gRNA) sequences targeting *Rlim* exon 5 (sense: 5′-gAT AAA TGT TAA CCG TAA CAA TGG-3′, antisense: 5′-gAA TCT GAA ATC ACC GCT GTT TGG-3′). Transfected cells were selected with 3 μg/mL puromycin for 48 hr and then plated at clonal density. Clones were analyzed by western blot and genomic DNA sequencing to confirm RNF12 knockout (Figure S1). To generate *Rlim* WT-internal ribosome entry site (IRES)-GFP (Rlim wt/y) and R575C-IRES-GFP (Rlim R575C/y) knockin ESCs cells, WT ESCs were transfected with vectors encoding guide RNAs targeting exon 5 in the *Rlim* gene (pBABED Puro U6 and pX335 containing 5′-GCA GGG CAG TCT TAT CTT CT-3′ and 5′-GTG GAA TTC TCA GAC AAC CAG-3′ sequences, respectively) and a donor vector containing RNF12 amino acids 84 to 600 followed by an IRES and EGFP. Transfected cells were selected as earlier and subjected to single-cell sorting. Single EGFP-positive cells were expanded and screened for EGFP expression via western blot, and knockin mutation was confirmed by genomic DNA sequencing.

### Western Blotting

Cells were harvested in lysis buffer containing 20 mM Tris (pH 7.4), 150 mM NaCl, 1 mM EDTA, 1% Nonidet P-40 (NP-40) (v/v), 0.5% sodium deoxycholate (w/v), 10 mM β-glycerophosphate, 10 mM sodium pyrophosphate, 1 mM NaF, 2 mM Na_3_VO_4_, and Roche Complete Protease Inhibitor Cocktail Tablets. 10–30 μg of cell lysate was loaded in SDS-PAGE gels and transferred to polyvinylidene fluoride (PVDF) membranes. Membranes were blocked with Tris buffered saline-tween 20 (TBS-T) 5% nonfat milk buffer (w/v). Primary antibodies used were RNF12 (Novus Biologicals), ERK1/2 (Santa Cruz Biotechnology), HA tag and REX1 (Abcam), ubiquitin (Dako), NANOG (ReproCELL), KLF4 (R&D Systems), DNMT3B (Imgenex), OCT3/4 (Santa Cruz Biotechnology), and FLAG (Sigma-Aldrich), all at 1:1,000. The following antibodies were raised in sheep by the Division of Signal Transduction Therapy, University of Dundee: RNF12 (S691D, third bleed, raised against residues 1–271 of mouse RNF12, DU49042) and RNF12 pSer212/214 (SA310, second bleed, raised against a QRRARpSRpSPEHRR phosphopeptide, where pS is phospho-serine). Both were used at 1 μg/mL after secondary antibody incubation, and membranes were subjected to chemiluminescence detection with Immobilon Western Chemiluminescent HRP (horseradish peroxidase) Substrate (Millipore) using a Gel-Doc XR+ System (Bio-Rad).

### Immunoprecipitation

20 μL of protein G agarose beads was washed three times with lysis buffer and incubated with 1 μg of mouse embryonic stem cell (mESC) lysate and 2 μg of antibody for 16–20 hr at 4°C. Beads were then washed three times with 500 mM lysis buffer, and immunoprecipitates were eluted with SDS sample buffer and boiled for 5 min for further analysis.

### Immunofluorescence

Cells were seeded in gelatin-coated coverslips and fixed with PBS 4% paraformaldehyde (PFA) (w/v). Fixed cells were permeabilized in PBS 0.5% Triton X-100 (v/v) for 5 min at room temperature (RT), blocked with PBS 3% BSA (w/v), and incubated with TUJ1 (Tubulin β 3, BioLegend) at 1:500, RNF12 antibody (S691D, third bleed, Division of Signal Transduction and Therapy, University of Dundee) at 1:200, or RNF12 antibody (Novus Biologicals) at 1:200 in blocking buffer for 2 hr at RT. Donkey anti-mouse Alexa 555, donkey anti-sheep Alexa 488, or donkey anti-mouse Alexa 488 (Thermo Fisher Scientific) were used as secondary antibodies at 1:500. Actin red 555 reagent (Thermo Fisher Scientific) was used for actin staining, and Hoechst (Sigma-Aldrich) at 1:10,000 was used for DNA staining. Cells were mounted using Fluorsave reagent (Millipore). Images were acquired in a Zeiss 710 confocal microscope and processed using ImageJ (NIH) and Photoshop CS5.1 software (Adobe). Neurite outgrowth was estimated by average outgrowth per cell, calculated as the ratio between neurite outgrowth length and total nuclei number, and quantified using the NeuriteTracer (Pool et al., 2008) and Cell Counter plugins in ImageJ (NIH). TUJ1(+) signal area per cell was determined as the ratio between the TUJ1(+) area and the Hoechst(+) area and quantified using the measurements tool in Volocity (PerkinElmer).

### *In Vitro* Ubiquitylation Assays

DyLight 800 maleimide (Thermo Fisher Scientific) Ub-IR^800^ was generated as described (Kumar et al., 2015). For ubiquitylation reactions, 1.5 μg of REX1 or SMAD7 were incubated with a 20 μL mix containing 0.1 μM UBE1, 0.05 μM E2 (UBE2D1, UBE2E2, UBE2W, or 28 other E2s tested), and 140 nM RNF12 (all from the Division of Signal Transduction Therapy, University of Dundee); 2 μM Ub-IR^800^; 0.5 mM Tris(2-carboxyethyl)phosphine (TCEP) (pH 7.5), and 5 mM ATP (both from Sigma-Aldrich); 50 mM Tris-HCl (pH 7.5); and 5 mM MgCl_2_ for 30 min at 30°C. Reactions were stopped with SDS sample buffer and boiled for 5 min. Samples were loaded in 4%–12% Bis-Tris gradient gels (Thermo Fisher Scientific). Gels were then scanned using an Odyssey CLx Infrared Imaging System (LI-COR Biosciences) for detection of fluorescently labeled ubiquitylated proteins. After scanning, proteins were transferred to PVDF membranes and analyzed via western blot.

### Cell-Based Ubiquitylation Assays

Modified haloalkane dehalogenase (HALO)-tagged tandem ubiquitin binding element (HALO-TUBE) beads were produced as described (Emmerich et al., 2013). For REX1 ubiquitylation assays, mESCs were transfected with REX1 and RNF12 expression vectors and cultured for 48 hr. Cells were extracted in lysis buffer supplemented with 100 mM iodoacetamide (Sigma-Aldrich) and 2 mg of protein were incubated with 20 μL of HALO-TUBE beads for 4 hr at 4°C. Beads were then washed with 500 Mm NaCl lysis buffer, eluted with SDS sample buffer, and boiled for 5 min. The presence of ubiquitylated proteins was analyzed via western blot.

### E2 Discharge Assays

UBE2D1 discharge assays were performed as described (Plechanovová et al., 2012). UBE2D1-ubiquitin thioester was prepared by incubating 100 μM UBE2D1 with 0.2 μM UBE1 (Division of Signal Transduction Therapy, University of Dundee), 100 μM ubiquitin (Sigma), 3 mM ATP and 0.5 mM TCEP (pH 7.5) (both from Sigma-Aldrich), 5 mM MgCl_2_, 50 mM Tris (pH 7.5), and 150 mM NaCl for 20 min at 37°C. Reaction was stopped by depleting ATP with 4.5 U/mL apyrase (New England Biolabs) for 10 min at RT. Then, 40 μM UBE2D1-ubiquitin was incubated with 280 nM RNF12 and 150 mM L-lysine in a buffer containing 50 mM Tris (pH 7.5), 150 mM NaCl, 0.5 mM TCEP, and 0.1% (v/v) NP-40 at RT. Reactions were stopped with nonreducing SDS loading buffer and analyzed via SDS-PAGE. Gels were stained with Coomassie staining (InstantBlue reagent, Expedeon) and scanned in an Odyssey CLx Infrared Imaging System (LI-COR Biosciences), and the protein signal was quantified using Image Studio software (LI-COR Biosciences). Reaction rates were determined by extrapolating protein signals in a standard curve of known concentrations of UBE2D1-ubiquitin conjugate and plotting concentration over time.

### RNA Extraction and qPCR

Total RNA extraction was performed by a column-based system (Omega) and then subjected to reverse transcription using iScript reverse transcriptase (Bio-Rad) according to the manufacturer’s guidelines. qPCR reactions were carried out using SsoFast EvaGreen Supermix (Bio-Rad) in a CFX384 real-time PCR system (Bio-Rad). The ΔΔCt method was used to express the relative RNA levels in the samples, and GAPDH expression was analyzed as a loading control. Data were analyzed in Excel software (Microsoft) and plotted in GraphPad Prism v.6.00 software (GraphPad). Primers used are listed in Table S1.

### Data Analysis

Pooled data are presented as mean ± SEM of at least three biological replicates unless stated in the figure legend. Statistical significance was determined using ANOVA followed by Tukey’s post hoc test or Student’s t test, and significant differences were considered when p < 0.05. For western blots, densitometric analysis was performed using Image Lab software (Bio-Rad). HA-REX1 half-life was determined by adjusting data to a one-phase decay nonlinear fit in GraphPad Prism.

## Supplemental Information

Document S1. Figures S1–S5 and Table S1

Document S2. Article plus Supplemental Information
