# Supplementary material for: RNF12 X-Linked Intellectual Disability Mutations Disrupt E3 Ligase Activity and Neural Differentiation
Source: Cell Rep. 2018 May 8;23(6):1599–611. doi: 10.1016/j.celrep.2018.04.022 (PMC5976579; doi:10.1016/j.celrep.2018.04.022)
Supplement: Document S1. Figures S1–S5 and Table S1 [file mmc1.pdf]

Cell Reports, Volume *23*

Supplemental Information

RNF12 X-Linked Intellectual Disability Mutations Disrupt E3 Ligase Activity and Neural Differentiation

Francisco Bustos, Anna Segarra-Fas, Viduth K. Chaugule, Lennart Brandenburg, Emma Branigan, Rachel Toth, Thomas Macartney, Axel Knebel, Ronald T. Hay, Helen Walden, and Greg M. Findlay

SUPPLEMENTARY DATA

Supplementary Table 1: Primer sequences, related to Experimental procedures

**Gene**	**Forward (5' to 3')**	**Reverse (5' to 3')**
Nanog	CTCATCAATGCCTGCAGTTTTTCA	CTCCTCAGGGCCCTTGTCAGC
Fgf5	GCTGTGTCTCAGGGGATTGT	CACTCTCGGCCTGTCTTTTC
Klf4	ACACTTGTGACTATGCAGGCTGTG	TCCCAGTCACAGTGGTAAGGTTTC
Dnmt3b	CTGGCACCCTCTTCTTCATT	ATCCATAGTGCCTTGGGACC
Sox1	TTCCCCAGGACTCCGAGGCG	GCTGTGTGCCTCCTCTGCGG
Pax6	GGACTTCAGTACCAGGGCAACC	GCATCTGAGCTTCATCCGAGTC
Ascl1	TCTCCTGGGAATGGACTTTG	GGTTGGCTGTCTGGTTTGTT
Brachyury	TCCCGAGACCCAGTTCATAG	TTCTTTGGCATCAAGGAAGG
Sox17	TATGGTGTGGGCCAAAGACGAA	AACGCCTTCCAAGACTTGCCTA
GAPDH	CTCGTCCCGTAGACAAAA	TGAATTTGCCGTGAGTGG

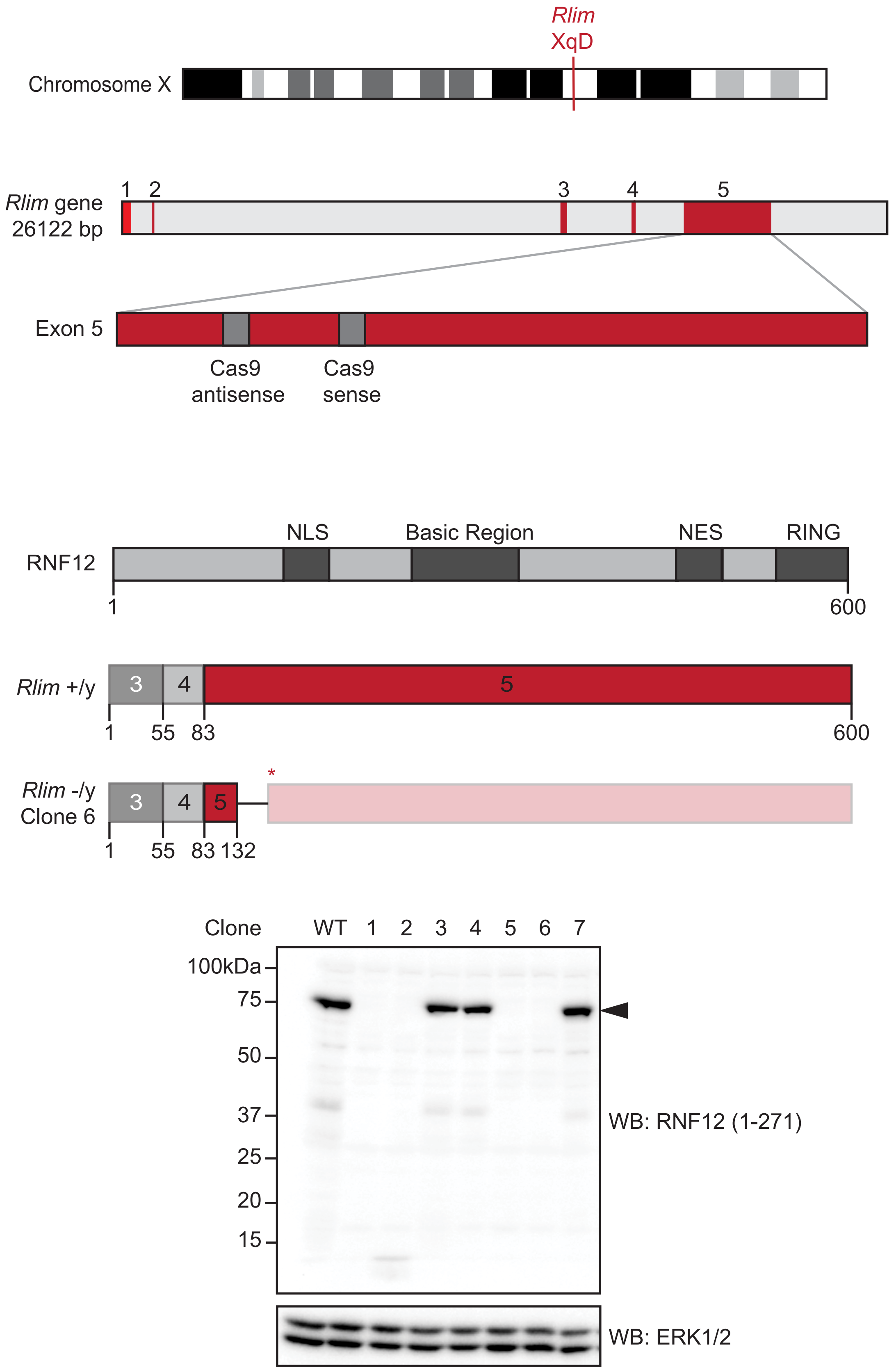

Figure S1. RNF12/Rlim CRISPR/Cas9 gene knockout strategy (related to Figure 1).
Paired CRISPR/Cas9 guide RNAs were designed to target exon 5 of the *Rlim* gene on Chromosome X. Genomic DNA sequencing analysis of the isolated clone 6 predicts a truncated mRNA/protein of 132 amino acids. RNF12 immunoblot analysis does not detect a protein product in resulting *Rlim* -/y ESC line.

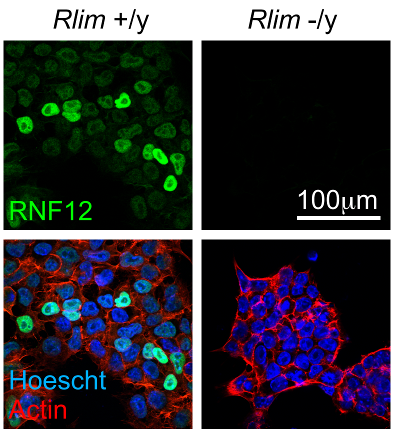

Figure S2. Endogenous RNF12 protein is localised to the nucleus (related to Figure 2).
Rlim +/y and -/y ESCs were analysed via immunofluorescence and confocal microscopy.

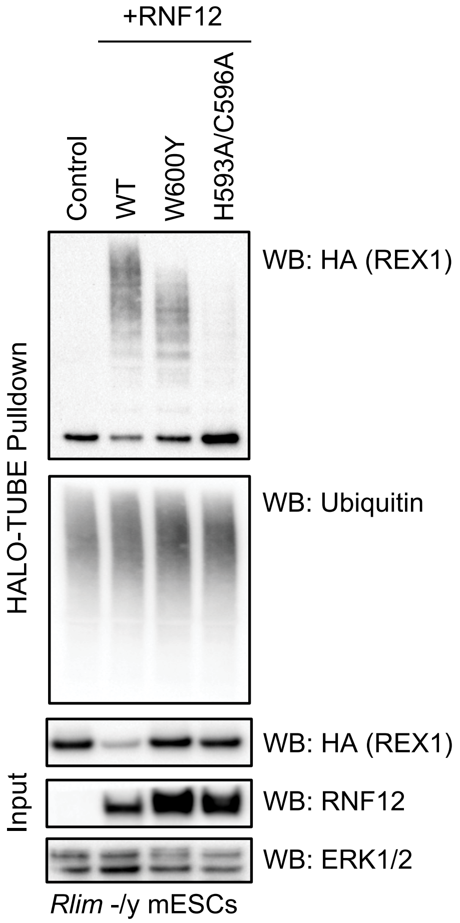

Figure S3. RNF12 catalytic activity is required for REX1 ubiquitylation and degradation (related to Figure 3).

Rlim -/y ESCs were transfected with HA-REX1 and wild-type RNF12 or the indicated catalytic mutants. Ubiquitylated proteins were captured using HALO-TUBE resin and HA-REX1 ubiquitylation determined by immunoblotting. Total ubiquitin levels are shown as a control. HA-REX1, RNF12 and ERK1/2 levels in cell lysates were determined by immunoblotting.

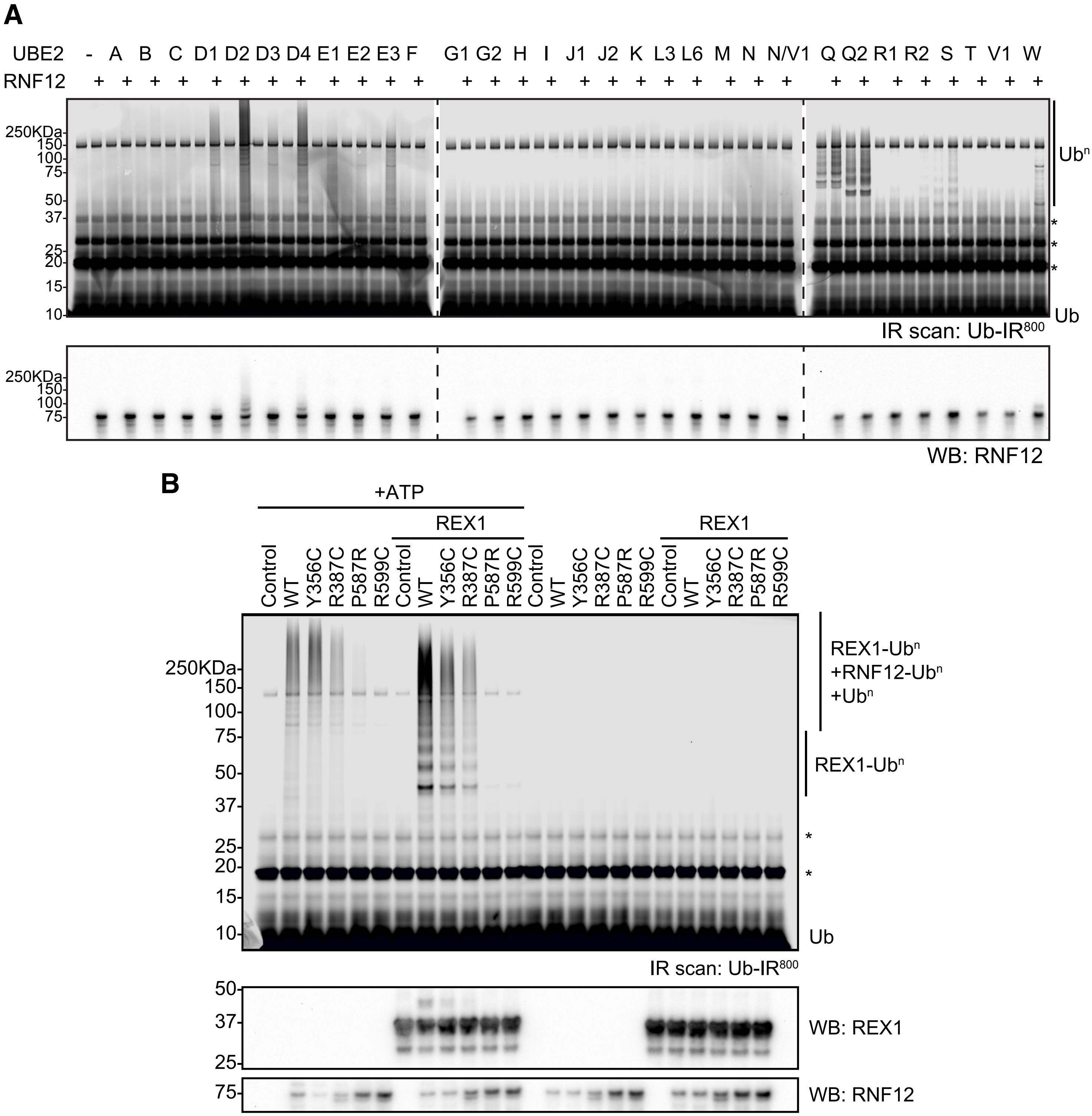

Figure S4. In vitro characterisation of RNF12 catalytic activity (related to Figure 4).
(A) Analysis of RNF12 ubiquitylation activity in combination with a panel of 31 E2 ubiquitin conjugating enzymes. Infra-red scan (Ub-IR^{800}) shows labelled fluorescent ubiquitin chains (Ub^{n}). RNF12 levels were determined by immunoblotting. * = non-specific fluorescent band. (B) In vitro REX1 ubiquitylation by WT or XLID mutant RNF12 proteins. Infra-red scan (Ub-IR^{800}) shows labelled fluorescently labelled ubiquitylated proteins. REX1 and RNF12 levels were determined by immunoblotting. Specific ubiquitylated REX1 (REX1-Ub^{n}) and RNF12 (RNF12-Ub^{n}) signals are indicated. * = non-specific fluorescent band.

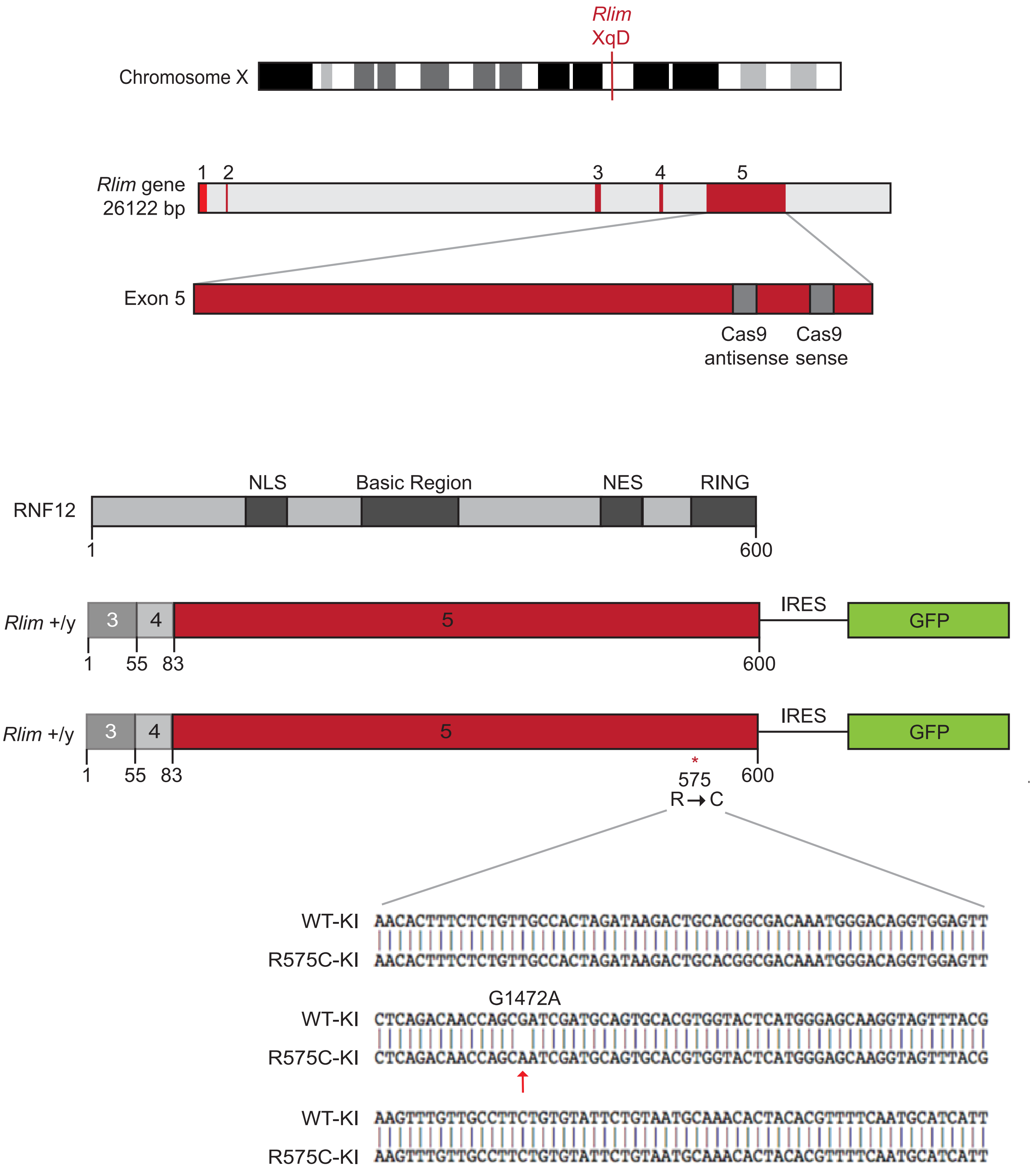

Figure S5. RNF12/*Rlim* WT and R575C CRISPR/Cas9 knock-in strategy (related to Figure 5).
Paired CRISPR/Cas9 guide RNAs were designed to target exon 5 of the *Rlim* gene on Chromosome X in order to replace endogenous RNF12 with either RNF12 WT or R575C fused to IRES-EGFP. Genomic DNA sequencing of *Rlim* WT-KI and R575-KI ESCs confirmed the presence of wild-type RNF12 or introduction of a single nucleotide substitution resulting in R575C mutation, respectively.
